# 798. Life-years Gained among non-Hispanic Black and White Men who have Sex with Men in the United States with Improvements in the HIV Care Continuum A Simulation Modeling Study

**DOI:** 10.1093/ofid/ofac492.058

**Published:** 2022-12-15

**Authors:** Katherine M Rich, Krishna Reddy, Aimalohi Ahonkhai, Fatma Shebl, Ankur Pandya, Elena Losina, Kenneth Freedberg, Emily P Hyle

**Affiliations:** Harvard Medical School, Boston, Massachusetts; Massachusetts General Hospital, Boston, Massachusetts; Vanderbilt University Medical Center, Nashville, Tennessee; Massachusetts General Hospital, Boston, Massachusetts; Harvard T.H. Chan School of Public Health, Boston, Massachusetts; Harvard Medical School; Brigham and Women's Hospital; Boston University School of Public Health, Boston, Massachusetts; Massachusetts General Hospital, Harvard Medical School, Boston, Massachusetts; Massachusetts General Hospital, Boston, Massachusetts

## Abstract

**Background:**

Structural barriers and racism result in inequities across the HIV care continuum. We quantified the magnitude of racial disparities in life expectancy (LE) and the potential impact of interventions to improve the HIV care continuum among non-Hispanic Black and White men who have sex with men (MSM) in the US.

**Methods:**

Using the validated CEPAC microsimulation HIV model, we projected LE among non-Hispanic Black MSM (BMSM) and White MSM (WMSM). We estimated average age at HIV infection (BMSM: 26.8y, WMSM: 35.0y) and time from infection to diagnosis (BMSM: 3.4y, WMSM: 3.0y) using US race-stratified data from the Centers for Disease Control and Prevention (CDC) (Table 1). To account for differences in the HIV care continuum, we calibrated input parameters to race-specific estimates of: 1) the proportion of time that MSM with diagnosed HIV are retained in care (BMSM: 75.2%, WMSM: 80.6%), and 2) the % of MSM with diagnosed HIV who attain virologic suppression (VS; BMSM: 82.0%, WMSM: 91.2%). To account for increased risk of non-HIV-related mortality, we adjusted race-stratified life tables for smoking. We then projected LE from age 15 in five scenarios: 1) status quo HIV care (2019 estimates of HIV testing, VS, and retention in care), 2) earlier diagnosis (via annual testing), 3) improved retention in care (95% retention via reduced loss to follow-up), 4) improved VS (95% VS among MSM in care), and 5) a combined strategy (annual testing, 95% retention, 95% VS).

**Results:**

Among MSM in status quo HIV care, we projected LE from age 15 to be 52.2y (BMSM) and 58.5y (WMSM), a difference of 6.3 years (Figure 1). With annual testing, BMSM would gain 0.6 life-years (LY), and WMSM would gain 0.3 LY compared with status quo care. Improving retention in care to 95% would result in a gain of 1.4 LY for BMSM and 1.0 LY for WMSM. BMSM would gain 1.1 LY if VS increased to 95% among those in care, whereas WMSM would gain 0.3 LY. BMSM would gain 3.4 LY (LE from age 15: 55.6y) and WMSM 1.6 LY (LE from age 15: 60.1y) in the combined strategy.

**Conclusion:**

Equity-focused solutions that specifically target investment in HIV care for Black MSM will be critical to reduce disparities in HIV care outcomes and improve LE.

**Disclosures:**

**Krishna Reddy, MD, MS**, UpToDate, Inc.: Author **Aimalohi Ahonkhai, MD, MPH**, Bryan Allen Events LLC for Gilead: Advisor/Consultant|ViiV: Advisor/Consultant.

**Figure:**
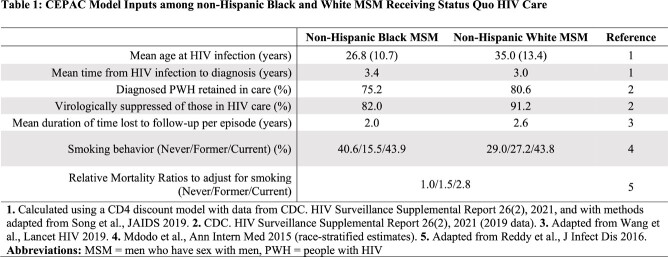

**Figure 1.**
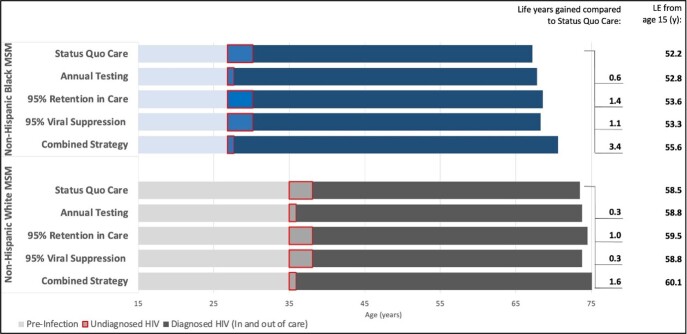
Model Outcomes Showing the Average Age at HIV Diagnosis and Life Expectancy of non-Hispanic Black and White MSM by HIV Care Scenario

